# Abdominal aortic aneurysm volume and relative intraluminal thrombus volume might be auxiliary predictors of rupture—an observational cross-sectional study

**DOI:** 10.3389/fsurg.2023.1095224

**Published:** 2023-05-05

**Authors:** I. Koncar, D. Nikolic, Z. Milosevic, N. Bogavac-Stanojevic, N. Ilic, M. Dragas, M. Sladojevic, M. Markovic, A. Vujcic, N. Filipovic, L. Davidovic

**Affiliations:** ^1^ Faculty of Medicine, University of Belgrade, Belgrade, Serbia; ^2^ Clinic for Vascular and Endovascular Surgery, Clinical Center of Serbia, Belgrade, Serbia; ^3^ Research and Development Center for Bioengineering BioIRC, Kragujevac, Serbia; ^4^ Faculty of Engineering, University of Kragujevac, Kragujevac, Serbia; ^5^ Belgrade-Faculty of Pharmacy, Department of Medical Biochemistry, Belgrade, Serbia

**Keywords:** AAA, volume, risk prediction, ILT, RRED, PWS

## Abstract

**Objectives:**

The study aimed to identify differences and compare anatomical and biomechanical features between elective and ruptured abdominal aortic aneurysms (AAAs).

**Methods:**

Data (clinical, anatomical, and biomechanical) of 98 patients with AAA, 75 (76.53%) asymptomatic (Group aAAA) and 23 (23.46%) ruptured AAA (Group rAAA), were prospectively collected and analyzed. Anatomical, morphological, and biomechanical imaging markers like peak wall stress (PWS) and rupture risk equivalent diameter (RRED), comorbid conditions, and demographics were compared between the groups. Biomechanical features were assessed by analysis of Digital Imaging and Communication in Medicine images by A4clinics (Vascops), and anatomical features were assessed by 3Surgery (Trimensio). Binary and multiple logistic regression analysis were used and adjusted for confounders. Accuracy was assessed using receiving operative characteristic (ROC) curve analysis.

**Results:**

In a multivariable model, including gender and age as confounder variables, maximal aneurysm diameter [MAD, odds ratio (OR) = 1.063], relative intraluminal thrombus (rILT, OR = 1.039), and total aneurysm volume (TAV, OR = 1.006) continued to be significant predictors of AAA rupture with PWS (OR = 1.010) and RRED (OR = 1.031). Area under the ROC curve values and correct classification (cc) for the same parameters and the model that combines MAD, TAV, and rILT were measured: MAD (0.790, cc = 75%), PWS (0.713, cc = 73%), RRED (0.717, cc = 55%), TAV (0.756, cc = 79%), rILT (0.656, cc = 60%), and MAD + TAV + rILT (0.797, cc = 82%).

**Conclusion:**

Based on our results, in addition to MAD, other important predictors of rupture that might be used during aneurysm surveillance are TAV and rILT. Biomechanical parameters (PWS, RRED) as valuable predictors should be assessed in prospective clinical trials. Similar studies on AAA smaller than 55 mm in diameter, even difficult to organize, would be of even greater clinical value.

## Introduction

Natural history of abdominal aortic aneurysms (AAAs) is progressive dilation until eventual rupture. The asymptomatic and insidious nature of this disease frequently prevents timely diagnosis, which is nowadays accomplished through national screening programs organized in certain countries. Open or endovascular repair of AAA has been substantially improved, providing low-risk preventive procedures to patients. However, early mortality of up to 2% for endovascular repair, 5% after open repair, and 20% rate of late reintervention after endovascular repair are precluding liberal use of AAA repair, and the threshold for repair is based on maximal aneurysm diameter (MAD) (1, 2). To optimize the risk–benefit ratio, it is useful to assess the risk of rupture. There are concerns about the validity of measuring the sensitivity to reveal disease progression by only MAD assessment (3). Aneurysm volume and thrombus thickness are associated with aneurysm growth or rupture; however, contrary to MAD, they are not widely accepted as parameters for clinical decision-making (4–6).

Finite-element analysis (FEA) may be used to more accurately estimate forces, like peak wall stress (PWS), acting on the aneurysm wall (7–10). Rupture risk equivalent diameter (RRED) may be used to compare biomechanical estimates among the patient groups (10). This parameter reflects the average aneurysm size that experiences the same estimates as the individual case. Subtracting the MAD from RRED can be used to compare among patient groups independently of the actual diameter (size). Such a biomechanical test is still not accepted in clinical practice even though PWS, as calculated with FEA, was significantly higher in ruptured AAAs than in intact AAAs across multiple studies and in one meta-analysis (11). Another systematic review concluded that although FEA is frequently applied in research, the methodology has not been standardized for AAA, and its technical limitations have only marginally improved. In addition, for using such a method, at least basic education of physicians in biomechanics and FEA is necessary (11, 12).

The aim of this study was to assess rupture risk prediction of comorbid conditions, patients’ demographics, maximal aneurysm diameters, and other morphological characteristics and to compare them with biomechanical imaging markers (PWS, RRED).

## Methods

The study was designed and presented in this paper according to Strobe's recommendations (13). It was performed at the university clinic from January 01, 2015, to January 01, 2016, and it was approved by the local ethical committee.

The observed outcome was the rupture of AAA. The rupture was defined if multidetector computed tomography (MDCT) examination showed AAA with visible loss of wall integrity and/or surrounding hematoma.

Two hundred and eighty-eight consecutive patients with AAA greater than 40 mm in diameter, both asymptomatic and ruptured, were included in the observational cross-sectional study. These patients underwent MDCT (slice thickness 0.625 mm). In our institution, MDCT is indicated in patients with more complex aneurysms (short neck, peripheral occlusive disease) or in those that are candidates for endovascular repair due to advanced age, comorbid conditions, or hostile abdomen. Operative treatment was indicated according to contemporary clinical practice guidelines, and treatment results were not part of this study.

Patients were excluded from the study in case of an inflammatory aneurysm (thickening of the aortic wall with a well-defined halo and an irregular external margin between the aneurysm and surrounding tissue) or low-quality images due to the low contrast volume in aortic lumen precluding biomechanical or morphological analysis. Also, patients operated on only according to ultrasonography examination were excluded. Two hundred and eighty-eight consecutive patients with AAAs of maximum diameters ≥40 mm were admitted to our institution during the study period. Out of them, 180 patients were excluded from the study due to the following reasons: lack of MDCT examination (133), presented with symptoms and no sign of rupture on MDCT (35), presented with inflammatory aneurysms (3), and low-quality images because of low contrast volume in the aortic lumen (9) that were difficult or impossible to analyze. Out of 288 patients considered for the study, 98 (34.02%) patients fulfilled the inclusion criteria and were included in the study.

Included patients were divided into two groups: patients with asymptomatic AAA (aAAA) and patients with ruptured AAA (rAAA).

Diagnostic criteria are as follows: AAA was considered asymptomatic if diagnosed in an elective setting, with no sudden and severe pain, and if ultrasound and MDCT did not show any irregularities in the aneurysm wall morphology. Ruptured AAA was considered any aneurysm revealed in an emergency setting, with sudden abdominal or back pain with MDCT signs of retroperitoneal hematoma. As explained above, patients with symptoms and no signs of rupture were excluded since they did not belong to any of the two groups.

Data regarding patients’ comorbidities and demographics were collected prospectively by a previously determined questionnaire.

Images of MDCT delivered in a DICOM (Digital Imaging and Communication in Medicine) file were analyzed by a single operator using available software—3Surgery (Trimensio) for morphological analysis and A4clinics (VASCOPS GmbH, Graz, Austria) for the morphological and biomechanical assessment.

*Morphological data* obtained by standard center lumen line analysis in the 3Surgery working station (Trimensio, Unites States) were MAD, neck diameter and length, aneurysm and iliac length, aneurysm diameter, and angulations. Angulations were calculated through angle measurements and by using the shape index—the ratio between the two different diameter measurements (shortest and longest ones) on the axial cross section (14). These measurements were made by common techniques also used in seizing and planning for endovascular procedures. *Morphological data* derived from the analysis in A4Clinics software were total AAA volume (TAV in cm^3^), ILT volume (in cm^3^), and maximal ILT thickness (in mm). These parameters were automatically calculated by the software. Since the ILT volume could depend on the total aneurysm volume, the relative intraluminal thrombus (rILT) volume was calculated, expressing the ILT volume in percentages: ILT volume  × 100/total aneurysm volume. *Biomechanical data* obtained from A4Clinics were PWS (in kPa) and RRED (in mm).

RRED was introduced by Gasser et al. to translate biomechanical rupture risk values into equivalent diameters of the average aneurysm patient with the same risk of rupture (based on epidemiologic and biomechanical data) (9, 10). Both parameters, PWS and RRED, are automatically calculated by A4Clinics software. To exclude the influence of pressure on RRED, values of boundary condition were the same for all patients. The FEA model was pressurized by the mean arterial pressure (MAP; 1/3 systolic pressure 2/3 diastolic pressure), which predicted the mechanical stress (force per area) in the wall of the aneurysm. This pressure was predefined as 120/80 (mean 92.3 mmHg).

As previously published, apart from geometry and arterial pressure, an FEA model requires constitutive descriptions of the wall and the ILT (10, 15). A constitutive description is a mathematical model of biomechanical properties, which relates stress and strain (deformation) and/or describes the strength of the tissue. The FEA models used in the present analysis considered isotropic constitutive descriptions for the ILT and the aneurysm wall. An isotropic constitutive model is a common approximation for aneurysm tissue and assumes that the tissue's mechanical properties do not depend on the orientation; i.e., the stress–strain responses of circumferential and longitudinal strips of tissue are identical.

To reduce selection bias, all subsequent AAAs were included in the analysis. However, a substantial number of AAA patients did not undergo an MDCT examination, which was the reason for their exclusion. The decision to perform or not MDCT was exclusively clinically driven, and the authors of this study had no influence on it.

## Statistical analysis

Data were entered into a customized database and analyzed using IBM SPSS Statistics, Version 20.0 (IBM Corp. 2011). We considered *P* values <0.05 as statistically significant.

Differences in continuous variables between the groups were analyzed by Student's *t*-test for normally distributed variables and by the Mann–Whitney *U* test for variables with the non-Gaussian distribution. Group differences for categorical variables were examined by the chi-square test, and univariate associations were evaluated by Spearman's correlation analysis.

Odds ratios (ORs) were calculated using binary logistic regression analysis to determine whether anatomical, morphological, and biomechanical parameters had any potential for predicting aneurysm rupture. Independent association of examined parameters (with high practical importance and availability) with aneurysm rupture was tested using multiple logistic regression analysis. Adjustments were made to correct the influence of confounder variables (gender and age). Age was entered as continuous and gender as a categorical (1, female; 0, male) variable.

The accuracy of the examined parameters was assessed using receiving operative characteristic (ROC) curve analysis. Statistically significant parameters were combined based on their practical importance and availability, the curve for this model was plotted, and the area under the ROC curve (AUC) was presented as C statistics from the analysis. By using the Hosmer and Lemeshow rule for logistic models, the discriminative abilities of the model were classified according to their AUC values as poor (0.5 ≤ AUC < 0.7), acceptable (0.7 ≤ AUC < 0.8), excellent (0.8 ≤ AUC < 0.9), or outstanding (AUC ≥ 0.9) (16).The optimal cut-off (cut-off with the highest Youden index − sensitivity + specificity) and correct patient classifications were calculated for examined parameters.

Data are shown as mean ± SD for normally distributed continuous variables, as median and quartile values for non-normally distributed variables, and as absolute and relative frequencies for categorical variables.

## Results

There were 75 (76.53%) asymptomatic (Group aAAA) and 23 (23.46%) ruptured AAAs (Group rAAA) with an average age of 70.6 ± 8.22 years. Comorbid conditions and demographic characteristics of patients in both groups are compared and presented in Table 1.

**Table 1:** Cardiovascular risk factors in asymptomatic and ruptured AAAs.

Comorbid condition	aAAA group, *N*=75 (%)	rAAA group, *N*=23 (%)	*p*
Mean age (years)	70 ± 7.9	71.4 ± 8.74	0.20
Male/female	63/12 (84/16)	18/5 (78.37/21.73)	0.524
Hypertension	60 (80)	15 (65.2)	0.564
Hyperlipoproteinemia	15 (20)	3 (13.04)	0.424
Statin therapy	9 (12)	2 (8.69)	0.217
Diabetes	6 (8)	2 (8.69)	0.230
Coronary artery disease	37 (49.33)	8 (18.4)	0.053
Previous PCI or CABG	21 (28)	16 (15.53)	0.111
Atrial fibrillation	9 (12)	7 (6.79)	0.176
COPD	15 (20)	5 (20.73)	0.229
Renal insufficiency	9 (12)	8 (7.76)	0.775
PAOD	8 (10.66)	7 (6.79)	0.855
Previous vascular operation	3 (4)	2 (1.94)	0.472
Carotid disease	15 (20)	6 (5.82)	0.153
Aneurysm in other arterial segments	3 (4)	1 (0.97)	0.198

**Table:** 

Comorbid condition	Study group, *N*=98 (%)	Non-study group, *N*=241 (%)	*p*
Mean age (years)	70.6 ± 8.2	71.4 ± 8.74	0.433
Male/female	81/17 (84/16)	214/27 (88.8/11.2)	0.048
Hypertension	75 (76.5)	206 (85.5)	0.498
Diabetes	8 (8.16)	25 (10.4)	0.300
Coronary artery disease	45 (45.91)	57 (23.7)	0.000
Atrial fibrillation	9 (12)	7 (6.79)	0.176
COPD	20 (20.4)	36 (14.9)	0.014
Renal insufficiency	9 (12)	8 (7.76)	0.775
PAOD	8 (10.66)	7 (6.79)	0.855
Previous vascular operation	3 (4)	2 (1.94)	0.472
Carotid disease	15 (20)	6 (5.82)	0.153
Aneurysm in other arterial segments	3 (4)	1 (0.97)	0.198

AAAs, abdominal aortic aneurysms; PCI, percutaneous coronary intervention; CABG, coronary artery bypass grafting; COPD, chronic obstructive pulmonary disease; PAOD, peripheral arterial occlusive disease.

Variables were compared by the chi-square test.

The differences in anatomical, morphological, and biomechanical parameters between the two groups are presented in Table 2 (only statistically significant parameters are presented).

**Table 2:** Significantly different anatomical, morphological, and biomechanical parameters between asymptomatic and ruptured AAAs.

Parameter	Total	aAAA	rAAA	*p*
MAD (mm)	66.59 ± 18.76	60.82 ± 15.49	79.30 ± 19.3	<0.001^a^
Total aneurysm volume (cm^3^)	201.0 (146.9–201.0)	187.4 (134.7–238.7)	337.4 (203.8–482.1)	<0.001^b^
Lumen volume (cm^3^)	91.8 (66.7–128.0)	87.4 (61.3–117.4)	130.7 (87.5–211.0)	<0.001^b^
ILT volume (cm^3^)	77.0 (39.7–138.3)	67.4 (36.8–111.0)	149.2 (84.4–297.0)	<0.001^b^
rILT volume (%)	39.68 (29.38–50.37)	37.12 (28.00–45.40)	45.31 (37.70–54.40)	0.027^b^
PWS (kPa)	224.1 ± 69.6	212.9 ± 64.5	269.7 ± 72.27	0.001^b^
RRED (mm)	56.99 ± 22.23	53.52 ± 18.90	71.41 ± 29.60	0.002^a^
Mean wall stress (kPa)	110.90 ± 24.06	108.44 ± 22.34	124.78 ± 29.18	0.020^a^
Mean stress in ILT (kPa)	7.15 ± 1.25	7.00 ± 1.28	7.86 ± 0.96	0.016^a^
Shape index in the neck zone^c^	0.75 ± 0.17	0.77 ± 0.16	0.68 ± 0.21	0.040^a^
Distance from lower renal to aortic bifurcation (mm)	116.38 ± 21.65	113.62 ± 19.19	126.20 ± 27.18	0.022^a^
Maximal ILT thickness (mm)	20.58 ± 11.04	18.61 ± 9.69	28.10 ± 13.07	<0.001^a^

AAAs, abdominal aortic aneurysms; rAAA, ruptured abdominal aortic aneurysms; ILT, intraluminal thrombus; MAD, maximal aneurysm diameter; PWS, peak wall stress; RRED, rupture risk equivalent diameter; rILT, relative intraluminal thrombus volume: ILT volume × 100/total aneurysm volume.

^a^ Data are expressed as mean ± SD and compared by Student’s *t*-test.

^b^ Values are expressed as medians and quartile values and compared by the Mann–Whitney *U* test.

^c^ Shape index demonstrates the level of angulation.

Regression analysis was performed to explore the association between rILT volume with other examined parameters. Values of rILT correlated with MAD in both groups, slightly higher in the rAAA group (*ρ* = 0.462; *p* = **0.035)** compared to the aAAA group (*ρ* = 0.245; *p* = **0.041**) (Bold significance are *p* < 0.05). On the other side, rILT correlated with total aneurysm volume (*ρ* **= **0.296; *p* = **0.011)**, neck diameter (*ρ* **= **0.309; *p* = **0.009)**, and neck length (*ρ* **= **0.294; *p* = **0.012)** in the aAAA group but not in the rAAA group for TAV (*ρ* **= **0.390; *p* = 0.081), aneurysm neck diameter (*ρ* **= **−0.168; *p* = 0.493), and aneurysm neck length (*ρ* **= **−0.071; *p* = 0.771) (Bold significance are *p* < 0.05).

We used binary logistic regression to determine whether anatomical, morphological, and biomechanical parameters had any potential for the prediction of AAA rupture. Unadjusted analysis showed that low values of the shape index in the neck zone (OR** **=** **0.560, *p* = 0.046) were associated with AAA rupture. In contrast, high values of RRED, MAD, PWS, mean wall stress, TAV, ILT volume, rILT volume, lowest renal to aortic bifurcation distance, and maximal ILT thickness increased AAA rupture probability. Data are presented in Table 3.

**Table 3:** Logistic univariate regression analysis for anatomical, morphological, and biomechanical predictors of AAA rupture.

Parameters	OR (95% CI)	*p*
RRED	1,031 (1,009–1,054)	0.007
MAD	1.062 (1.041–1.082)	<0.001
PWS	1.011 (1.004–1.018)	0.003
Mean wall stress	1.027 (1.003–1.051)	0.028
Total aneurysm volume	1.006 (1.003–1.010)	<0.001
Lumen volume	1.010 (1.003–1.016)	0.004
ILT volume	1.011 (1.005–1.018)	<0.001
rILT volume	1.041 (1.003–1.079)	0.032
Neck length	0.976 (0.932–1.021)	0.293
Neck diameter	1.020 (0.944–1.102)	0.620
Shape index in the neck zone	0.56 (0.003–0.950)	0.046
Shape index in the aneurysm zone	0.031 (0.01–4.416)	0.170
Shape index in the iliac zone	0.380 (0.041–3.552)	0.396
Lowest renal to aortic bifurcation distance	1.030 (1.003–1.059)	0.032
Maximal ILT thickness	1.084 (1.029–1.141)	0.002

AAAs, abdominal aortic aneurysms; rAAA, ruptured abdominal aortic aneurysms; OR, odds ratio; CI, confidence interval; MAD, maximal aneurysm diameter; PWS, peak wall stress; RRED, rupture risk equivalent diameter; TAV, total aneurysm volume; rILT, relative intraluminal thrombus volume.

All variables are continuous.

Adjusted logistic regression analysis was performed to explore rILT volume, TAV, and MAD predictive abilities for AAA rupture. In a multivariable model, including gender and age as confounder variables, all previously mentioned parameters continued to be significant predictors of AAA rupture (MAD: OR** **= 1.063; TAV: OR** **=** **1.006; rILT: OR** **=** **1.039; PWS: OR** **=** **1.010; and RRED: OR** **=** **1.031). Data are presented in Table 4.

**Table 4:** Multivariable logistic regression analysis for anatomical, morphological, and biomechanical predictors of AAA rupture.

	OR (95% CI)	*p*
**Adjusted for gender and age**		
MAD	1.063 (1.042–1.085)	<0.001
TAV	1.006 (1.003–1.010)	0.001
rILT volume	1.039 (1.002–1.078)	0.038
PWS	1.010 (1.003–1.018)	0.009
RRED	1.031 (1.008–1.054)	0.006

AAAs, abdominal aortic aneurysms; OR, odds ratio; CI, confidence interval; MAD, maximal aneurysm diameter; PWS, peak wall stress; RRED, rupture risk equivalent diameter; TAV, total aneurysm volume; rILT, relative intraluminal thrombus volume.

Gender is a categorical variable. All variables are continuous.

Finally, we explored the diagnostic abilities of RRED, MAD, PWS, rILT volume, and TAV for AAA rupture (Table 5). Although the AUC corresponding to MAD (AUC = 0.790) was the highest among the obtained AUC values, the correct classification at the optimal cut-off value of 68 mm was 75%. TAV showed acceptable diagnostic accuracy with 79% correct classification, but the accuracy for rILT was poor. At the optimal cut-off value of 41.3 derived from the curve, only 60% of subjects were correctly classified based on rILT. Correct classification by PWS and RRED was 73% and 55%, respectively, at optimal cut-off values of 249.8 kPa for PWS and 46 mm for RRED. Furthermore, we explored whether the combination of MAD, TAV, and rILT improves the prediction of AAA rupture on existing parameters. The AUC for the model with combined parameters indicated an acceptable ability for AAA rupture prediction (Table 4). The combination of three parameters increased the correct patient classification to 82%. The results of ROC and C analyses for discriminating asymptomatic from ruptured AAA are presented in Table 5.

**Table 5:** Results of ROC and C analyses for discriminating asymptomatic from ruptured AAAs.

	AUC 95% CI	Cut-off values	Correct classification, %
MAD, mm	0.790 (0.726–0.854)	68	75
PWS, kPa	0.713 (0.593–0.832)	249.8	73
RRED, mm	0.717 (0.590–0.844)	0.46	55
TAV, cm^3^	0.756 (0.626–0.886)	310.4	79
rILT volume %	0.656 (0.529–0.783)	41.3	60
MAD + TAV + rILT	0.797 (0.687–0.908)	0.05^a^	82

^a^ Probability cut-off for AAA rupture.

AAAs, abdominal aortic aneurysms; ROC, receiving operative characteristic; AUC, area under the ROC curve; CI, confidence interval; MAD, maximal aneurysm diameter; PWS, peak wall stress; RRED, rupture risk equivalent diameter; TAV, total aneurysm volume; rILT, relative intraluminal thrombus volume.

## Discussion

Our study has shown that biomechanical parameters (PWS and RRED) are greater in rAAA and some morphological aneurysm features like TAV, rILT, and MAD. Combined into logistic models, MAD, rILT, and TAV might have good rupture risk prediction values, better than MAD, PWS, or RRED alone.

The rationale for AAA repair is based on the ratio between the procedure and rupture risk. Contrary to procedure risk, rupture risk is difficult to assess accurately. MAD as a common criterion has been challenged in the last two decades. Important progress was made using FEA with PWS as the main outcome variable (8). Further improvement potentiated analysis of aortic tissue strength and its computed estimation that enabled calculating the ratio between the wall stress and wall strength expressed through the potential rupture index (17). Gasser et al. went even further with their RRED and translated biomechanical information into our surgical language of maximal diameter (9).

A recent systematic review identified 1,503 potentially relevant articles that assessed biomechanical imaging markers and their potential association with AAA growth or rupture (12). The authors concluded that published studies had confounding bias between groups due to baseline differences in aneurysm diameter, did not report basic characteristics (demographics, comorbidity) of the included patients, and FEA methodology has not been standardized (12). PWS was significantly higher in ruptured than in intact AAAs across multiple studies, as shown in the previous meta-analysis.

In addition, nowadays, it is still difficult to include biomechanical analysis of AAA rupture risk in common clinical practice. It requires not only dedicated software but also educated personnel capable of performing and translating information into clinical practice, standardized research studies to determine thresholds for intervention, education of vascular surgeons in FEA and biomechanics, the inclusion of bioengineers as a part of the multidisciplinary aortic team, etc.

Previous studies rarely described the demographics of included patients (12). None of the comorbid conditions in our study could be used for the rupture prediction model since there were no differences in this regard between the groups. Smoking habit is still very present in Serbia, so both groups had a very high rate of smokers. MAD, PWS, and RRED as predictors of rupture were adjusted for gender and age.

MAD is used in our clinical practice to present aneurysm size and consequently rupture risk. Basically, if centerline analysis is used, such a measure is gained from one cross section of the aneurysm sac with improved accuracy. However, such a measure does not provide sufficient information about the aneurysm magnitude. For these purposes, aneurysm volume might better represent its real magnitude and consequently its growth. In our study, TAV was a predictor of aneurysm rupture independent of aneurysm diameter. Previous papers showed that an aneurysm is not always growing at the level of the longest diameter (4, 5).

ILT has its role in aneurysm evolution and rupture development. It was shown that ILT influences the histology of the aneurysm wall and promotes rupture by inducing hypoxia or weakening the aneurysm wall (6, 18). Since ILT volume might depend on aneurysm size, we expressed the volume of ILT as a percentage of total aneurysm volume through rILT volume. In this manner, we avoided the influence of MAD on ILT volume, and rILT volume was an independent predictor of aneurysm rupture.

Finally, we combined MAD as the main parameter (as we are used to it), TAV (as a better measure of aneurysm magnitude), and rILT (as a better expression of ILT extent) in the logistic model that showed its predictive value of AAA rupture under the ROC curve, which is comparable to parameters gained from biomechanical analysis. RRED has an advantage since it calculates wall stress and tissue properties, while the three parameters included in the model only represent anatomy and morphology. On the other side, MAD, TAV, and rILT volume can be measured easily in everyday practice and could also be used in the follow-up. Further analysis, publication, and education of surgeons should be organized to make biomechanical testing available in everyday practice.

The concept of biomechanical rupture risk assessment was proposed more than 20 years ago and is still under investigation. It was not accepted in common clinical practice due to probably different reasons. Measuring the MAD is very simple and familiar to all vascular physicians. No matter that such a measurement is also not standardized and very simplified, underestimating the complexity of the pathology of the aneurysm may lead to AAA growth and rupture. Measuring biomechanical features of a particular aneurysm is neither complicated nor time-consuming (it takes less than 30 min); however, it requires dedicated software and an understanding of the concept that rupture occurs when biomechanical forces overcome wall strength. Finally, we need more convincing data that this concept works. In the recent systematic review, only 300 patients were included in seven comparative studies with moderate to high risk of bias (21). The present study might support clinicians to measure aneurysm volume and even rILT volume during follow-up of small aneurysms or those of longer diameters unsuitable for treatment and use it in decision-making together with MAD as additional information.

## Limitations

Even though data were collected prospectively, this is a cross-sectional study. Also, a significant number of patients were excluded from the study due to the lack of MDCT images or their inadequate quality. The majority of excluded patients were from the 4A group, and no differences in demographic and comorbid parameters were noticed between the excluded and included patients (this analysis was not included in the Results section due to limited space). There were significantly fewer patients with rAAA in this study. The reasons are multiple. rAAA is a rare condition, patients with rAAA are not always undergoing MDCT, or the examination has been done in another institution and patients were sent without the electronic version of the examination. Contrast timing is not perfect in unstable or bleeding patients with low image quality and potential to perform analysis. Patients with ruptured AAA had greater values of MAD; however, this was included in multivariate analysis. No sensitivity analysis has been performed in our study. All measurements in the study were made by one, although a very experienced, person. For the measurement of anatomical and morphological parameters, the author has more than 1,000 planned endovascular aneurysm repair (EVAR) cases, while for performing measurements of biomechanical variables, a dedicated understanding and validation were performed with authors of A4Clinic software. Previous papers showed good inter- and intraobserver variability (22) when using A4Clinics software. FEA models introduce numerous modeling assumptions and cannot completely reflect the biomechanics of the real aneurysm in a particular patient, considering calcifications or other morphological irregularities of the aneurysm wall (19). Likewise, 10 patients with ruptured aneurysms were excluded from the study since it was not possible to differentiate the aortic wall from the surrounding hematoma. Also, the presented biomechanical data in this study must always be seen in relation to the specific modeling assumptions. In our study, we focused on boundary conditions of arterial pressure of 120/80 mm Hg; however, exposing aneurysm morphology and geometry to different boundary conditions might show different results. This assumption should be included in future studies (20). Finally, there were few patients with aneurysms smaller than 55 mm in diameter. Future studies should focus more on these patients since these aneurysms are also prone to rupture, and identification of those at higher risk would support clinical decision-making and prevent eventual unexpected rupture.

## Conclusion

Based on our results, in addition to maximal aneurysm diameter, other important predictors of rupture are total aneurysm volume and relative volume of intraluminal thrombus, which might be used during aneurysm surveillance. Biomechanical parameters (PWS, RRED) as valuable predictors should be used in clinical practice for patients included in clinical trials where their estimation will be standardized. Future studies on small aneurysms are needed to improve clinical decision-making and prevent unexpected rupture.

## Data Availability

The raw data supporting the conclusions of this article will be made available by the authors, without undue reservation.

## What this paper adds

This paper shows that when assessing the risk of abdominal aortic aneurysm (AAA) rupture, in addition to diameter, the magnitude of the aneurysm should be presented with the total aneurysm volume. In addition, the volume of intraluminal thrombus expressed relatively to the total aneurysm volume should be considered. These aneurysm characteristics are possible to measure routinely nowadays and could be used during surveillance, especially in patients at risk of intervention. On the other side, biomechanical parameters are important and should be systematically incorporated into clinical studies and common practice in the future. The results of this study could apply to aneurysms with diameters longer than 55 mm since there were a low number of aneurysms with shorter diameters.
